# Pluripotency-associated genes in human nasopharyngeal carcinoma CNE-2 cells are reactivated by a unique epigenetic sub-microenvironment

**DOI:** 10.1186/1471-2407-10-68

**Published:** 2010-02-25

**Authors:** Jun-Xia Cao, Yu-Xin Cui, Zi-Jie Long, Zhong-Min Dai, Ji-Yan Lin, Yi Liang, Fei-Meng Zheng, Yi-Xin Zeng, Quentin Liu

**Affiliations:** 1 State Key Laboratory of Oncology in South China, Cancer Center, Sun Yat-sen University, 650 Dongfeng Road, Guangzhou 510060, PR China; 2 The Jackson Laboratory, 600 Main Street, Bar Harbor, Maine 04609, USA; 3 Department of Pathology, University of Cambridge, Hills Road, Cambridge BS2 0QQ, UK; 4 Department of Hematology, Third Affiliated Hospital, Sun Yat-sen University, 600 Tianhe Road, Guangzhou 510630, PR China; 5 College of Life Sciences, Zhejiang University, 388 Yuhangtang Road, Hangzhou 310058, PR China; 6 Department of Emergency, First Affiliated Hospital, Sun Yat-sen University, 58 Zhongshan 2nd Road, Guangzhou 510060, PR China

## Abstract

**Background:**

There is increasing evidence that cancers contain their own stem-like cells, and particular attention has been paid to one subset of cancer-stem cells termed side population (SP). Stem cells under normal physical conditions are tightly controlled by their microenvironment, however, the regulatory role of the microenvironment surrounding cancer stem cells is not well characterized yet. In this study we found that the phenotype of SP can be "generated" by macrophage-like cells under conditioned culture. Furthermore the gene regulation pathway involved in cellular reprogramming process was investigated.

**Methods:**

The selection and identification of SP in 50 CNE-2 single cell clones were performed by flow cytometry. The transwell assay and immunofluorescence staining were used to measure migration and cancer stem cell characters of non-SP single clone cells cultured with conditioned medium respectively. The subtraction suppression hybridization (SSH) technique and northern blotting analysis was applied to explore the pluripotency-associated genes under a unique epigenetic sub-microenvironment.

**Results:**

Among 50 clones, only one did not possess SP subpopulation while others did. The non-SP cells induced by macrophage-like cells showed more aggressive characters, which increased cell migration compared with the control cells and showed some fraction of SP phenotype. These cells expressed distinguished level of pluripotency-associated genes such as ADP-ribosylation factor-like 6 interacting protein (ARMER), poly (rC) binding protein 1 (PCBP1) and pyruvate dehydrogenase E1-β subunit (PDHB) when subjected to the environment.

**Conclusion:**

To our knowledge, this is the first study to demonstrate that non-SP single-clone cells can be induced to generate a SP phenotype when they are cultured with conditioned medium of macrophage-like cells, which is associated with the reactivation of pluripotency-associated genes.

## Background

The hypothesis of cancer stem cell (CSC) suggests that neoplastic clones are maintained exclusively by a small subpopulation of cells that give rise to phenotypically diverse cancer cells [1-3]. CSCs were first identified in 1990s in hematological malignancies, mainly acute myelogenous leukemia (AML) and also in other subtypes like AML M0, M1, M2, M4, and M5, chronic myeloid leukemia (CML), acute lymphoblastic leukemia (ALL), and multiple myeloma [4,5]. This subset of cancer cells was also found in solid tumors in the breast, brain, lung, prostrate, testis, ovary, stomach, colon, skin, liver and pancreas [6-11]. Thus, there is overwhelming evidence that certain pathways which regulate or maintain the extensive proliferative and self-renewal potential of the tumor clone are present in CSC.

It is known that niches control stem cell function, thus it may seem counterintuitive that CSCs would be located within these regulatory microenvironments. Since this question was first raised in 2005 [12], soon in 2007 evidence was shown that the vascular niches in brain tumors are abnormal and contribute directly to the generation of CSCs and tumor growth [13]. Therefore, CSCs might arise from normal stem cells that have acquired mutations that enable them to escape from niche control. Alternatively, deregulation of extrinsic factors within the niche might lead to uncontrolled proliferation of stem cells and tumorigenesis [14]. The fate of CSCs depends upon aberrant niche microenvironments to some extent, thus these niches might represent targets for the treatment of cancer. We paid particular attention to the source of CSCs and possible regulatory pathway in nasopharyngeal carcinoma (NPC) microenvironment herein.

NPC is a malignancy of the head and neck region that arises from the epithelial cells that cover the surface and line of the nasopharynx. This disease was initially reported in 1901, and characterized clinically in 1922 [15]. It is endemic in many geographical regions, including Southern China and Southeast Asia, where the observed incidence rates range from 15 to 50 per 100,000 persons. Previous studies have reported that squamous cell carcinoma (former WHO type 1) accounts for approximately 25% of all NPC; whereas undifferentiated carcinoma (former WHO type 3) accounts for 95% of all cases in high incidence areas [15-18]. The management of recurrent cervical lymph node metastases in NPC after radiation and chemotherapy is a radical surgery of the lymph nodes of the neck with postoperative brachytherapy. The salvage surgical procedure for persistent or recurrent neck disease shows a 5-year control rate of 66% and a 5-year actuarial survival of 38% [18]. Thus, the control of the differentiation and metastases may be the key to understanding this carcinoma. Our study combining the NPC CSCs with microenvironment aimed to address this key question.

Some markers and phenotype of CSCs were similar to that of normal neural stem cells. For example, they express CD133 and nestin in brain cancer, CD44 in breast cancer and CD34 in leukemia [4,5,7,8]. Recent evidences demonstrate that ABCG5 and USP22 may be used as cancer stem cell marker [19,20]. On the other hand, a character of stem cells termed side population (SP) can be identified due to the cellular exclusion of Hoechst 33342 dye. The SP cells have been isolated by flow cytometric analysis from various types of adult tumor tissue where they possess stem cell activity, including lung adenocarcinoma, gastrointestinal cancers, head and neck squamous cell carcinoma, ovarian cancer, thyroid cancer, and hepatocellular carcinoma [21-27]. Here, we performed our work on human NPC CNE-2 cells, of which SP phenotype revealed several stem cell properties [28], to investigate whether NPC CSCs are tightly regulated by the immediate microenvironment, to evaluate the differential gene expression profile of the CSCs with microenvironment, and to find more specific stem cell marker for NPC CSCs.

In this study, we tried to identify cancer non-SP cell clone from human NPC cancer cell line CNE-2. Such non-SP cell clone was isolated and cultured with macrophage-like cells. We found that conditioned culture could induce generation of SP cells by the non-SP cells derived from both NPC and neuroblastoma cell lines. Moreover, we evaluated the cell cycle and migration ability of condition cultured non-SP cells, which cells showed G1/S arrest and enhanced motility compared with control. We further identified the differential gene profile in condition cultured non-SP cells by the subtraction suppression hybridization (SSH).

## Methods

### Cell lines and reagents

Human acute monocytic leukemia cell line THP-1, human nasopharyngeal cancer cell line CNE-2 and human neuroblastoma cell line SK-N-CH were obtained from American Tissue Culture Collection (Rockville, MD, USA).

Antibodies including CD133 and CD133-PE were purchased from Miltenyi Biotech (Bergisch Gladbach, Germany). All the other reagents were purchased from Sigma (St Louis, MO, USA) unless otherwise noted.

### Single clone selection

Cells were seeded in a 24-well plate with 1 cell/ml and single cell clones were isolated from wells with only a solitary colony. Selecting 50 single cell clones were subjected to expansion culture until sufficient amounts of cells were obtained. The 50 single cell clones were detected by the flow cytometric analysis of SP and non-SP distribution respectively. One CNE-2 single clone is named CNE-2-2 with little SP phenotype.

### Macrophage-conditioned medium

THP-1 cells at a density of 0.5 × 10^6^/ml were cultured in RPMI 1640 (Invitrogen, San Diego, CA, USA) supplemented with 10% FBS (Hyclone, Logan, UT, USA) and 20 ng/ml phorbol 12-myristate 13-acetate (PMA) for 48 h at 37°C in a humidified atmosphere of 5% CO_2 _incubator. After nonadherent cells were discarded, remaining cells were washed twice in PBS and once in RPMI 1640 with 15-min incubation at 37°C. These PMA induced macrophage-like cells were cultivated for 24 h with fresh RPMI 1640 containing 10% FBS (8 ml per 75-cm^3 ^flask). The supernatant after culture was subsequently filtered and used as the macrophage-conditioned medium.

### Cell culture

NPC Cells were cultured in a 25-cm^2 ^tissue culture flask (1.2 × 10^4^cells/cm^2^) in RPMI 1640 supplemented with 10% FBS, 50 U/ml penicillin and 50 μg/ml streptomycin for 24 h. Cells were washed with PBS and the conditioned medium produced by PMA-activated THP-1 cells was added. After indicated cultivation days, nonadherent cells were gently washed away with PBS and adherent cells were harvested with 0.05% trypsin-EDTA for test.

### Flow cytometry

To identify the SP cells in the cancer cells, we selected the single clone cells as described above. The cells were removed from the culture dish with trypsin/EDTA (Invitrogen) and resuspended at 10 cells per ml RPMI 1640 supplemented with 2% FBS, 50 U/ml penicillin and 50 μg/ml streptomycin. Cells were preincubated in a 1.5-ml Eppendorf tube for 90 min at 37°C with 2.5 μg/ml Hoechst 33342 dye (Invitrogen), either alone or in combination with 50 M verapamil, an inhibitor of some verapamil-sensitive ABC transporters [29]. Finally, the cells were counterstained with 1 μg/ml propidium iodide to label dead cells. Cells were analyzed in a FACSVantage fluorescence-activated cell sorter (Becton Dickinson). Propidium iodide-positive dead cells (<15%) were excluded from the analysis.

The non-SP Cells from the single clone detected by FACS were seeded into a 6-well plate at 1 × 10^5 ^cells/well and incubated with conditioned medium at 37°C. After 24 h of incubation, cells were collected with trypsin/EDTA. SK-N-CH cells were incubated with CD133-PE antibody for 10 minutes at 4°C before FACS analysis. CNE-2-2 single-cell suspension was fixed in ice-cold 70% ethanol at -20°C for 16 h, labeled with propidium iodide (50 μg/ml) for at least 15 min in dark and analyzed directly on a FACSCalibur flow cytometer (Becton Dickinson Immunocytometry Systems, San Jose, CA, USA).

### Immunofluorescence staining

Cells grown on cover slips were washed in cold PBS and fixed in 2% paraformaldehyde-PBS for 20 min and permeabilized in 0.5% Triton X-100 in PBS for 10 min at 4°C. The cells were incubated with 1% BSA for 30 min at room temperature (RT) to block nonspecific binding before the primary antibody reaction. Cells were incubated with the primary antibodies to CD133 for 1 h at RT, and then a specific FITC conjugated secondary antibody for 1 h. Cells were counterstained with DAPI (1 μg/μl), and examined using a fluorescence microscope (Olympus BX51).

### Transwell migration assay

CNE-2-2 cells were starved overnight in assay media (RPMI 1640 with 1% FBS). The top chamber of a 24-well transwell plate (Corning Inc.) was pretreated with 1% Matrigel™ (BD Biosciences, San Jose, CA, USA) in PBS and incubated 1 h at RT. Cells (10^5^/ml) were added to the top chambers of the plates. Media with or without activated macrophage-like cells was added to both chambers. After overnight incubation, top cells were removed and bottom cells were fixed and stained with DAPI (5 μg/ml) to visualize nuclei. The number of migrating cells in five fields was counted under fluorescence microscope, and the mean for each chamber was determined.

### Nucleic acid preparation

All of the cells were snap-frozen in liquid nitrogen and stored at -80°C. The tested cells were lysed in Trizol Reagent (Invitrogen, CA, USA), and total RNA was isolated according to the manufacturer's instructions. mRNA of the conditioned culture non-SP single cell clone and non-conditioned culture cells was purified from total RNA using the Oligotex mRNA Spin-Column kit (Qiagen, Hilden, Germany).

### Suppression subtractive hybridization (SSH)

SSH was performed between conditioned culture non-SP single cell clone and non-conditioned culture cells using the PCR-Select cDNA Subtraction Kit (Clontech, Palo Alto, CA) according to the manufacturer's recommendations. In the subtraction, conditioned culture non-SP single clone cells were used as a tester and non-conditioned culture single clone cells were used as a driver. Double-strand cDNA was synthesized from poly (A)+ RNA and blunted according to the kit manual. Two tester populations were created with different adaptors, but no adaptors with the driver.

After two cycles of hybridization, we carried out 28 cycles of primary PCR and 21 cycles of secondary PCR with DNA polymerase (Promega, Madison, WI, USA) on a GeneAmp 2400 thermal cycler (PE Biosystems, Foster City, CA, USA). To evaluate the efficiency of cDNA subtraction, we compared the expression of human β-actin (NP_001092) using the primers as denoted on Table 1 in subtracted and unsubtracted PCR products respectively. The subtracted fragments were then inserted into the T/A cloning vector pCR 2.1-TOPO (Invitrogen, CA, USA).

**Table 1:** Northern blot probe primers

Gene symbol		Primer sequence
ARMER	F-primer	CGGAGGGAGATAATCGCAGCACCAAC
	R-primer	GAAGCAGGTTGTGGACTTGTTGTCCC
PCBP1	F-primer	CGCCGGAATTGACTCCAGCTCTCC
	R-primer	CAGAAAGGGGTTATTGAGGGAACCTAC
PDHB	F-primer	CGGTGTCTGGCTTGGTGCGGAGAC
	R-primer	GAGTTTATAACCTGGTCAATGGCTTGC
FUBI	F-primer	TTTCAGATTCTAGAGTTATGTCCTC
	R-primer	ATAGAATGGGATGCTGTCTGGGAA
β-actin	F-primer	CGCGCTCGTCGTCGACAACGGC
	R-primer	CGTACATGGCTGGGGTGTTGAAGGTC

### Differential Screening

120 individual recombinant clones were picked and used as templates for PCR amplification using vector primers M13F and M13R. Each PCR product (about 1 μl) was transferred to a nylon membrane (Hybond-N, Amersham Pharamacia Biotech, Uppsala, Sweden). For denaturing purposes, the membrane was submerged for 2 - 3 min in turn on the three pieces of Whatman 3 MM paper saturated with 1.5 M NaCl and 0.5 M NaOH, 1.5 M NaCl and 0.5 M Tris-HCl (pH 8.0), 0.2 M Tris-HCl (pH 7.5) and 2 × SSC buffer, respectively. Subtracted and unsubtracted PCR products were used as probes labeling with digoxigenin-dUTP at 37°C overnight according to the DIG High Prime Labeling Kit protocol (Roche Molecular Biochemicals, Mannheim, Germany). The dot blot hybridization was performed overnight at 42°C using the DIG Easy Hyb system (Roche Molecular Biochemicals, Mannheim, Germany) following the manufacturer's instructions. The blots were washed at RT twice at low stringency (2 × SSC, 0.1% SDS) for 5 min and then at 68°C twice at high stringency (0.1 × SSC, 0.1% SDS) for 15 min. Hybridized probes were subsequently detected by alkaline phosphatase-conjugated anti-DIG antibody and the CSPD chemiluminescent detection system (Roche Molecular Biochemicals, Mannheim, Germany).

### Sequencing and Sequence analysis

Nucleotide sequences were compared with the GenBank using the BLASTn programs from the National Center for Biotechnology Information http://www.ncbi.nlm.nih.gov/BLAST/ and the BLASTx for the protein. Protein patterns were then identified by searching the PROSITE database http://www.expasy.ch/tools/ and Pfam database http://pfam.sanger.ac.uk/.

The DNA was sequenced from both strands on an ABI PRISM 377 Genetic Analyzer (PE Biosystems, Foster City, CA, USA).

### Northern blotting analysis

Digoxigenin-labelled DNA probes were applied for Northern blotting analysis. Oligonucleotide primers for the DNA probes were designed based on the known sequences (Table 1). The programs were as follows: 94°C for 5 min, followed by 35 cycles of 30 sec at 94°C, 30 sec at 56°C, and 90 sec at 72°C, and a final polymerization step at 72°C for 10 min, and then the PCR products were purified using QIAquick PCR Purification Kit (Qiagen, Hilden, Germany). The fragments were labeled with digoxigenin-dUTP as above.

Each set of total RNA was extracted from conditioned culture non-SP single clone cells and non-conditioned culture single clone cells described above. Total RNA was quantified on a spectrophotometer at 260 nm. Aliquots containing 10 μg of total RNA corresponding to each tissue were mixed with glyoxal and DMSO and heated at 50°C for 30 min and then placed on ice over 1 min. The treated RNAs were then separated through a 1% agarose (Seakem) gel. The intensity of ethidium bromide-stained rRNA bands was examined visually to ensure that equal amounts of total RNA were loaded onto the gels. The RNA was then transferred to a nylon membrane (Hybond-N, Amersham Pharamacia Biotech, Uppsala, Sweden), and UV cross-linked. Hybridization was performed overnight at 50°C, washed at RT twice at low stringency for 5 min and then at 55°C twice at high stringency for 15 min and were detected as above.

## Results

### Identification of SP cells in the CNE-2 single clones

**Figure 1:**
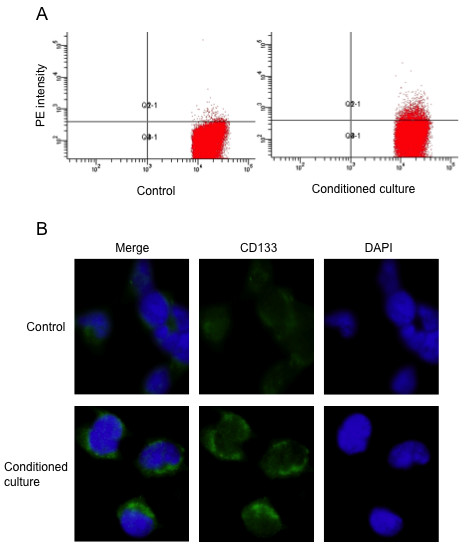
**FACS analysis of side population (SP) in the CNE-2 cell line labeled with Hoechst 33342**. (A and B) SP cells exist in CNE-2 single clone cells at a various percentage; (C) One clone contains little SP cell; (D) SP cells are increased by cultured with macrophage-like cells. The bottom panel shows the reduction of SP after treatment with 50 μM of verapamil. The data are representative of experiments over three times with similar results.

To determine whether different percentages of SP cells were contained in CNE-2 cells, we screened the CNE-2 cells by single clone selection. We stained the single clone cells respectively with Hoechst 33342, which is actively extruded by verapamil-sensitive ABC transporters. The SP cells from representative single clone CNE-2 ranged from 0.1% to 73.7% as estimated by FACS analysis (Fig. 1A, B and 1C). In each case, by definition, the number of SP cells was decreased greatly by treatment with verapamil (Fig. 1 bottom panel). We could detect little SP cells in just a single clone cell (Fig. 1C, clone 2). Thus, most of the CNE-2 cancer cell clones contained a small or big component of SP cells. Our study is focus on whether microenvironment might have an effect on the percentage of SP cell in CNE-2, i.e, whether it play a role in the cancer stem cell pool, so the further experiments were mainly performed the human CNE-2 single clone (namely CNE-2-2) with little SP phenotype.

We cultured the CNE-2-2 clone with conditioned medium and carried out FACS analysis for the presence of SP. As shown in Fig. 1D, the CNE-2-2 cell clone contained 9.8% SP cells after cultured with macrophage-like cells, whereas the control CNE-2-2 cells only contained 0.1% SP. The data therefore suggested that macrophage might be responsible for maintaining the CNE-2 cancer stem cell pool.

### Validation SP alteration in neuroblastoma SK-N-CH cells

**Figure 2:**
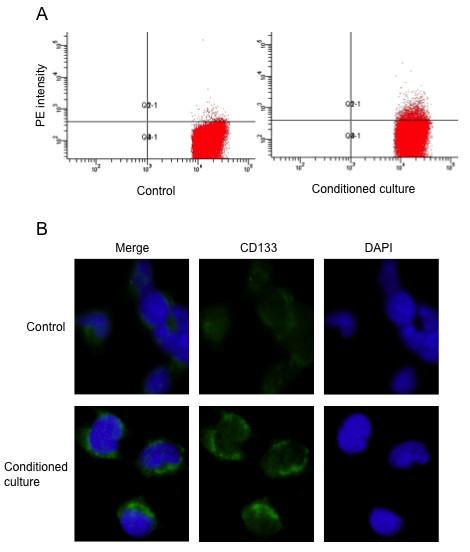
**Validation of SP cell fraction increase by cultured with macrophage-like cells in human neuroblastoma cell line SK-N-CH**. (A) Conditioned culture SK-N-CH cells show higher percentage of the CD133-PE population by FACS. (B) Immunofluorescence staining confirms the higher expression of CD133 (green). The images shown are representative of cells after cultivation for 24 hours. Nuclei were counterstained with DAPI (blue). Original magnification ×600.

To further confirm the above phenomena, we selected another cell line, the human neuroblastoma cell line SK-N-CH in which we could barely detect the presence of SP and the expression of CD133, one of the markers of neuroblastoma cancer stem cells. After cultured with activated macrophage medium, SK-N-CH cells were stained with CD133 antibody for FACS analyses. As shown in Fig. 2A, the condition cultured SK-N-CH cells contained 4.5% cancer stem cells, whereas the control SK-N-CH cells only contained 0.6%.

Furthermore, we examined the expression of CD133 on SK-N-CH cells in response to the conditioned culture induced by macrophage-like cells with immunofluorescence. The fluorescence density of CD133 positive cells was evidently stronger than that in control (Fig. 2B). These results allowed us to conclude that human macrophages did play a role in maintaining cancer stem cell pool at least in several solid tumors.

### G1-S cell cycle and migration of CNE-2 single clone cell affected by conditioned culture

**Figure 3:**
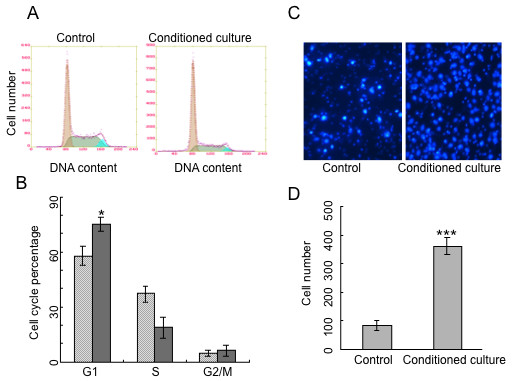
**Activated macrophage-like cells' medium inhibits G1-S transition and enhances migration in CNE-2 single clone cells**. (A) Cell cycle distribution of CNE-2 single clone cells after cultivation for 24 hours. (B) Percentage of G1, S and G2-M phases in CNE-2 single clone cells. Values are means ± SD (n = 3). * *P *< 0.01 vs control. (C) The migration of the CNE-2 single clone cells is enhanced by conditioned culture for 18 h in a transwell apparatus. Images shown are representative of three independent experiments. Nuclei were labeled with DAPI (blue), original magnification ×200. (D) Migration level is shown by quantified the migrated cells in 10 random fields per filter. Values are means ± SD (n = 3). *** *P *< 0.001 vs control.

Next, we analyzed cell cycle distribution in CNE-2-2 cells with conditioned medium. Cell cycle profiling of CNE-2-2 cells revealed that the conditioned culture induced reduction of the G1-S transition at 24 hours (Fig. 3A and 3B). By contrast, Control experiments showed no cell growth arrest features at 24 hours.

Following culture with activated macrophage-like cells, derived from human THP-1 cells, in a Transwell Boyden chamber system without cell-to-cell contact, CNE-2-2 cells resulted in a robust increase (>4 folds) in cell motility after 18 hours, whereas in control the normal culture conditions did not enhance the migration ability of CNE-2-2 cells (Fig. 3C and 3D). Taken together, these results clearly indicated that the functional components in macrophage-like cells responsible for cancer stem cell were complicated.

### Differential gene expression profile of conditioned culture CNE-2 single clone cell

The SP pool expansion, G1/S arrest and migration improvement in CNE-2-2 cells described above implied the cancer stem cell change in the cultured environment provided by macrophage-like cells. We further determined gene pathways involved during this process and studied the differential gene expression profiles between condition cultured and non-condition cultured CNE-2-2 cells by substractive suppression hybridization (SSH). About 120 clones were obtained from the SSH library enriched for the conditioned cultured CNE-2-2 cells transcripts. Following PCR using vector primers M13F and M13R and DNA dot-blot screening, 45 clones exhibiting expression differences were selected for DNA sequence analysis, which included 5 up-regulated (fold change >2) and 40 up-regulated (fold change >1.2) genes (Table 2).

**Table 2:** Categories of altered genes in CNE-2 single clone cells by macrophages-like cells during conditioned culture

Accession No.	Gene description	Alteration
NM_001012	Ribosomes This gene encodes a ribosomal protein that is a component of the 40S subunit.	Up
NM_002140.2	heterogeneous nuclear ribonucleoproteins (hnRNPs)	Up
NM_005174.2	Homo sapiens ATP synthase, H+ transporting, mitochondrial F1 complex, gamma polypeptide 1 (ATP5C1), nuclear gene encoding mitochondrial protein, transcript variant 2, mRNA	Up
NM_015937.3	Homo sapiens phosphatidylinositol glycan anchor biosynthesis, class T (PIGT), mRNA	Up
NM_018085.4	Homo sapiens importin 9 (IPO9)	Up
NM_000314.4	Homo sapiens phosphatase and tensin homolog (PTEN)	Up
NM_001033756.1	Homo sapiens vascular endothelial growth factor A (VEGFA), transcript variant 7, mRNA	Up
NM_003064.2	Homo sapiens secretory leukocyte peptidase inhibitor (SLPI)	Up
NM_002394.4	Homo sapiens solute carrier family 3 (activators of dibasic and neutral amino acid transport), member 2 (SLC3A2), transcript variant 3	Up
NM_005496.3	Homo sapiens structural maintenance of chromosomes 4 (SMC4), transcript variant 1	Up
NM_006145.1	Homo sapiens DnaJ (Hsp40) homolog, subfamily B, member 1 (DNAJB1)	Up
NM_002306.2	Homo sapiens lectin, galactoside-binding, soluble, 3 (LGALS3), transcript variant 1	Up
NM_020186.1	Homo sapiens ACN9 homolog (S. cerevisiae) (ACN9)	Up
NM_005347.2	Homo sapiens heat shock 70 kDa protein 5 (glucose-regulated protein,78 kDa) (HSPA5)	Up
NM_016639.1	Homo sapiens tumor necrosis factor receptor superfamily, member 12A (TNFRSF12A)	Up
NM_005348.3	Homo sapiens heat shock protein 90 kDa alpha (cytosolic), class A member 1 (HSP90AA1), transcript variant 2	Up
NM_194247.2	Homo sapiens heterogeneous nuclear ribonucleoprotein A3 (HNRNPA3)	Up
NM_004148.3	Homo sapiens ninjurin 1 (NINJ1)	Up
NM_002337.1	Homo sapiens low density lipoprotein receptor-related protein associated protein 1 (LRPAP1)	Up
NM_001003703.1	Homo sapiens ATP synthase, H+ transporting, mitochondrial F0 complex, subunit F6 (ATP5J), nuclear gene encoding mitochondrial protein, transcript variant 1	Up
NM_004048.2	Homo sapiens beta-2-microglobulin (B2M)	Up
NM_000146.3	Homo sapiens ferritin, light polypeptide (FTL)	Up
NM_001105079.1	Homo sapiens fibrosin (FBRS), mRNA	Up
NM_001002.3	Homo sapiens ribosomal protein, large, P0 (RPLP0) transcript variant 1	Up
NM_015134.2	Homo sapiens myosin phosphatase Rho interacting protein (MPRIP), transcript variant 1	Up
NM_004888.3	Homo sapiens ATPase, H+ transporting, lysosomal 13 kDa, V1 subunit G1 (ATP6V1G1)	Up
NM_015161.1	Homo sapiens ADP-ribosylation factor-like 6 interacting protein 1 (ARL6IP1)	Up
NM_001001973.1	Homo sapiens ATP synthase, H+ transporting, mitochondrial F1 complex, gamma polypeptide 1 (ATP5C1), nuclear gene encoding mitochondrial protein, transcript variant 1	Up
NM_001997.3	Homo sapiens Finkel-Biskis-Reilly murine sarcoma virus (FBR-MuSV) ubiquitously expressed (FAU)	Up
NM_006196.2	Homo sapiens poly(rC) binding protein 1 (PCBP1)	Up
NM_000925.2	Homo sapiens pyruvate dehydrogenase (lipoamide) beta (PDHB)	Up
NR_003286.1	Homo sapiens 18S ribosomal RNA (LOC100008588)	Up
BAG64005.1	unnamed protein product	Up
NW_001838014.1	Homo sapiens chromosome 10 genomic contig, alternate assembly	Up
NT_007592.14	Homo sapiens chromosome 6 genomic contig, reference assembly	Up
NW_001839064.2	Homo sapiens chromosome 7 genomic contig, alternate assembly	Up

**Figure 4:**
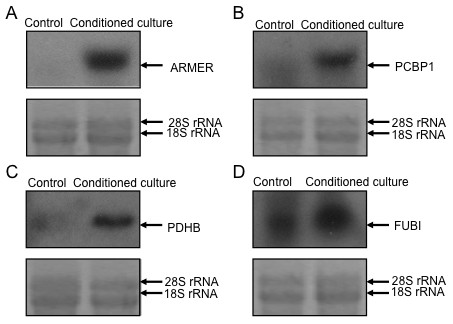
**Northern blotting analysis of SSH data**. Conditioned culture CNE-2 single clone cells show detectable higher mRNA transcription of ARMER (A), PCBP1 (B), PDHB (C) and FUBI (D) compared with control. The ethidium bromide-stained 28S/18S rRNA bands were used as a control for loading variation.

The SSH data was further validated by Northern blotting analysis. Results revealed that mRNAs of ADP-ribosylation factor-like 6 interacting protein (ARMER), poly(rC) binding protein 1 (PCBP1) and pyruvate dehydrogenase E1-β subunit (PDHB) were only expressed in conditioned culture CNE-2-2 (Fig. 4A, B and 4C), and the level of ubiquitin-like protein fubi and ribosomal protein S30 precursor (FUBI) mRNA transcript was higher in conditioned cultured cells compared with control (Fig. 4D).

## Discussion

In this study we have identified the non-SP cell clone in CNE-2 cells and found that conditioned culture non-SP cells were able to generate SP phenotype when microenvironment were present. Non-SP cells cultured with macrophage-like cells could not only produce heterologous descendent cells but also showed cell cycle arrest and higher cell motility. Furthermore we have determined the gene pathway involved during these processes by the SSH. To our knowledge, this is the first report to demonstrate the correlationship between non-SP cells and microenvironment in a tumor.

The impetus to CSC-theory has been only recently proposed due to the advances in stem cell biology although the concept of CSCs is not new. We showed here for the first time that non-SP cancer cells cultured with macrophage-like cells were able to generate SP phenotype probably by asymmetric division or reprogramming. In addition, by using different cell line (SK-N-CH) and cancer stem cell marker CD133, we have observed the consistent phenomena. It has been reported that brain CSCs are maintained within vascular niches [13]. Here we provided evidences that CSCs depended upon aberrant niche microenvironments indeed, hence these niches might represent targets for treatments of cancer. Further understanding of the fate of cancer stem cells interfered by their particular microenvironment may bring out a new era for cancer treatment.

A cancer stem cell is thought to be quiescent and should be in G0/G1 phase [30]. Non-SP cells cultured with macrophage-like cells could be arrested on G1/S thus slow down the cell cycle. It therefore indicated that the microenvironment had an effect on cell-cycle-specific change. On the other hand, the migration of the non-SP cells is enhanced through conditioned culture. Therefore, these results implied that non-SP cells showed more aggressive characteristics with response to microenvironment.

Comprehensive analysis of gene expression using SSH revealed distinct differences between conditioned culture and non-conditioned culture cells. A few genes have been found to be evidently up-regulated in conditioned cultured non-SP cells. Genes such as ARMER, PCBP1 and PDHB were only expressed in conditioned cultured non-SP cells, which can be validated by Northern blotting analysis. ARMER is an apoptotic regulator in the membrane of the endoplasmic reticulum [31], and was one of the most intensely up-regulated gene when cultured with the differentiated THP-1 conditioned media. Figure 3 reveals a large amount of apoptotic cells in the control and proportionally less in the conditioned culture population. Thus, the possible anti-apoptotic effect of ARMER may be induced when cultured with the conditioned medium. While PCBP1 plays a role in the protein-protein interaction network for human inherited ataxias and disorders of Purkinje cell degeneration [32]. PDHB is involved in tricarboxylic acid cycle of fibroblast [33]. We also found that FUBI mRNA transcript level was increased in conditioned cultured cells compared with control. Overexpression of FUBI leads to cell death, which can be halted by Bcl-2 or inhibition of caspases [29,34]. We have tried to compare our gene profile data with those of similar study on hepatocellular carcinoma, thyroid cancer and lung adenocarcinoma, but they are not in the same pattern suggesting the heterogenic nature of different malignancies. None of the up-regulated genes in non-SP cells found here have been documented to be involved in NPC. This might be partly explained by the different characteristics of non-SP cells and SP cells. The abundance of SP in CSCs from the same tumor contributes to distinct gene expression patterns, which has been often neglected by previous studies for many years.

## Conclusion

We have identified, for the first time, non-SP single clone cells can generate SP phenotype when they are cultured with macrophage-like cells. It should be noted that non-SP single clone cells derived directly from NPC CNE-2 cells would like to maintain non-SP characters, whereas non-SP cells sorted with FACS were inclined to gain SP. The underneath mechanism of the interesting phenomena is far yet clear. Further studies are needed to further pin-point the original source of the cancer stem cell in NPC and apply it for establishment a novel therapy of this malignancy.

## Competing interests

The authors declare that they have no competing interests.

## Authors' contributions

JXC, YXZ and QL designed and performed the study and wrote the manuscript. YXC, ZJL, ZMD, JYL, YL and FMZ also performed the migration, flow cytometry, subtraction suppression hybridization and northern blotting experiments, as well as assisted in revising it critically for important intellectual content. All authors read and approved the final manuscript.

## Pre-publication history

The pre-publication history for this paper can be accessed here:

http://www.biomedcentral.com/1471-2407/10/68/prepub
